# Comment on “Broadband Criticality of Human Brain Network Synchronization” by Kitzbichler MG, Smith ML, Christensen SR, Bullmore E (2009) PLoS Comput Biol 5: e1000314

**DOI:** 10.1371/journal.pcbi.1004174

**Published:** 2015-05-07

**Authors:** Simon Farmer

**Affiliations:** Sobell Department of Motor Neuroscience and Movement Disorders, Institute of Neurology, University College London, Queen Square, London, United Kingdom; John Radcliffe Hospital, United Kingdom

I wish to comment on a paper recently published in the *Journal of Neuroscience* [1] and relate this paper to one previously published in *PLOS Computational Biology* [2]. In [1] and [2] among other results, a power law relationship was discovered in a measure of magnetoencephalography (MEG) intra-areal synchronization: the distribution of phase-locking intervals (PLI). However, in [1] the authors also show that the same PLI power law measure cannot distinguish between human MEG and empty MEG scanner data, suggesting that the measure is vulnerable to artefact.

This is important because the first description of the PLI power law methodology, as well as its application to MEG and functional magnetic resonance imaging (fMRI) data, was published in *PLOS Computational Biology* [2]. The senior author of [2] is also an author of [1]. The results obtained with the PLI methodology published in [2] were presented as evidence for broadband criticality of human brain network synchronization because the PLI method (which applies a threshold to MEG/fMRI data and returns a power law) will also return a power law distribution for PLIs when applied to a model system of Kuramoto oscillators tuned to a critical phase transition. However, it should be noted in this regard that a recent modelling study indicates that power laws also emerge when PLI is applied to noncritical Kuramoto oscillators, and caution is needed when interpreting power laws derived from time series data passed through a threshold [3].

The paper published in *PLOS Computational Biology* [2] has received multiple citations, and the PLI methodology has been further applied to human neurophysiological data in other studies. From its use, claims have been made about changes in broadband criticality during disease states, e.g., epilepsy [4].

I am concerned that the results presented in [1] indicate that the methodology described in [2] may be unsound and therefore should not be used for inferring criticality of human brain network synchronisation. The presentation of the empty MEG scanner results in [1] is not sufficiently explicit in this sense, and it should have been made much clearer that the influential PLI power law methodology presented in [2] might be problematic.

Science of course moves forward through a process of exploration and correction, and the initial idea presented in *PLOS Computational Biology* [2] was exciting and innovative; however, the authors should reconsider the interpretation of their findings in light of the empty MEG scanner data. I feel it is therefore important to bring these papers and their conflicting results to the attention of the readers of *PLOS Computational Biology* and I would ask that the authors of [1,2] clarify the discrepancy in between their data sets and publish an erratum in *PLOS Computational Biology* if a conflict exists.

## References

[1] Shriki O, Alstott J, Carver F, Holroyd T, Henson RNA, Smith ML, et al (2013) Neuronal Avalanches in the Resting MEG of the Human Brain. J Neurosci 33: 7079–7090. 10.1523/JNEUROSCI.4286-12.2013 23595765 PMC3665287

[2] Kitzbichler MG, Smith ML, Christensen SR, Bullmore E (2009) Broadband criticality of human brain network synchronization. PLoS Comput Biol 5: e1000314 10.1371/journal.pcbi.1000314 19300473 PMC2647739

[3] Botcharova M, Farmer SF, Berthouze L (2012) Power-law distribution of phase-locking intervals does not imply critical interaction. Phys Rev E 86: 051920 23214827 10.1103/PhysRevE.86.051920

[4] Storch A, Hallmeyer-Elgner S, Bullmore ET, Gross T (2012) Failure of Adaptive Self-Organized Criticality during Epileptic Seizure Attacks PLoS Comput Biol 8: e1002312 10.1371/journal.pcbi.1002312 22241971 PMC3252275

